# Factors affecting adoption and use of M-commerce services among the customers in Saudi Arabia

**DOI:** 10.1016/j.heliyon.2022.e12532

**Published:** 2022-12-22

**Authors:** Mohammad Wasiq, Amar Johri, Prakash Singh

**Affiliations:** College of Administrative and Financial Sciences, Saudi Electronic University, Riyadh, Saudi Arabia

**Keywords:** M-Commerce services, Personal factors, Ease of doing factors, Economic factors, Covid-19 pandemic factors

## Abstract

M-commerce has the potential to change consumers’ shopping habits and establish itself as a significant commerce channel. People rely on digital devices more than ever before, and the growth in M-commerce predicts that mobile will become the preferred channel for online shopping soon. This study is aimed at examining the effect of personal factors, economic factors, ease of doing factors, and safety-related factors due to Covid-19 on the adoption and use of M-commerce services among customers in Saudi Arabia. The study is empirical and is based on survey responses from 340, M-commerce customers in Saudi Arabia. The questionnaire method was used to collect the data. ANOVA and bivariate regression analysis were used to evaluate the collected data. The results showed that four independent variables, namely, personal, economic, ease of doing, and safety factors during the Covid-19 pandemic, are significant predictors of the dependent variable, adoption and use of M-commerce services by the customers. These factors influence customers’ purchasing decisions when they use M-commerce services. The study also concluded that the frequency of using M-commerce has increased during the Covid-19 pandemic because of health, safety, and social distancing guidelines. One of the main limitation of the study is the few selective constructs for the research. The finding of the study will be beneficial to the customers to understand the significance of M-commerce services, especially during pandemic situations.

## 1 Introduction

The internet has changed the dimension of business [1, 2]. Dramatically shifting data from desktops to hand-held devices has encouraged users to purchase goods and services online [3]. Compared to other Middle Eastern countries, the exponential growth of mobile devices and wireless technologies provides excellent opportunities for customers to shop using their mobile phones. “Internet saturation is exceptionally high in Saudi Arabia, especially compared to other Middle Eastern countries.” Saudi Arabia is a country in the Middle East. The number of internet users among Saudis is nearly 20,831,695, which is about a 64.7% saturation rate” [4]. The trend has shifted from traditional to internet commerce, a revolutionary shift. Traditionally, electronic commerce has been used to purchase and sell products and services [5]. Electronic commerce is also considered buying and selling goods and services online. The tremendous growth of mobile equipment and wireless networks has given E-commerce a new shape. The new E-commerce version, known as M-commerce, is grabbing the customer’s attention. M-commerce is a subset of electronic commerce [6, 7, 8]. E-commerce implementation for the invention of the internet, while M-commerce implementation increased after the invention of smart devices and wireless networks.

E-commerce and M-commerce require different infrastructures. The growth of e-commerce was due to the rapid development of the Internet, which solves the global inter-networking problem and ensures that computers reliably communicate. At the same time, M-commerce was invented from private mobile communication systems. Private mobile communication companies offer various wireless media communication technologies ranging from worldwide to regional, such as Satellite technology, regional (3G), and Bluetooth technology [9, 10]. However, the objective is to facilitate consumer business development more effectively, proficiently, and rapidly.

M-commerce refers to wireless electronic commerce used for business through hand-held devices like smartphones, tablets, and PDAs. Mobile devices are considered personal diary that increases the number of mobile users.

According to the United Nations Conference on Trade and Development, M-commerce is described as the purchasing and selling of products and services via wireless hand-held devices [11]. An e-commerce application in a wireless setting, mainly via the internet, is known as mobile commerce [12].

For consumers, M-commerce allows them to conduct online transactions via mobile devices and access various personalized services at any time and location [13]. M-commerce allows merchants to reach their customers anytime and anywhere through a handheld or wireless devices and the internet. In contrast, E-commerce requires computers and the internet to reach customers. M-commerce allows customers to transmit various transactions such as financial transactions, bill payments, buying tickets, transferring funds, mobile banking, medical appointments, buying products, and receiving offers from private and government organizations by using a mobile device and accessing personalize services anytime from anywhere without the location restriction [14, 15]. Private and government organizations are making efforts to make mobile applications available in the marketplace. All these features have made M-commerce more popular among users.

Due to the Covid-19 pandemic, the lockdown period was challenging for the traditional retail sectors. The Covid-19 pandemic was taken as an opportunity by E-commerce and M-commerce organization. Significant growth has been seen in M-commerce and E-commerce organizations during the Covid-19 pandemic. Some E-commerce and M-commerce platforms connected to their respective online market claimed that since the Covid-19 crisis, average sales had increased by 200 per cent, average order value increased by 50 per cent, and apart from this, app installation spiked 400 per cent [16]. In this study, authors have identified these factors such as personal factors, economic factors, ease of doing factors, and safety factors to reveal the M-commerce user’s adoption during the COVID-19 pandemic. The country has imposed a complete lockdown due to the COVID-19 virus threat. It has shown that users’ M-commerce adoption increased in the country. Authors acknowledged that social distancing is a critical factor in using digital payment systems during the COVID-19 pandemic [17]. This has motivated us to study the adoption and use of M-commerce services among customers in Saudi Arabia.

Several research has been done on M-commerce adoption and use. On the other hand, further research is needed to understand the M-commerce adoption behaviour [18]. This issue is more complex in developing countries. Authors found that users from developing countries have different motivations and regulatory orientations to use M-commerce from those in developed countries [19]. The covid-19 pandemic has distressed the social, economic, personal, and safety factors. Due to safety measures during the Covid-19 pandemic, global crises have been seen in traditional retail stores. This has given M-commerce and E-commerce companies a competitive advantage over traditional retail stores.

The present study attempts to identify the factors that affect the adoption and use of M-commerce services in Saudi Arabia. In addition to personal, economic, and ease of doing factors, this paper will also focus significantly on Covid 19 factors on M-commerce adoption and use.

M-commerce services are less frequent than purchasing goods and services from nearby retail shops or malls. There are multiple economic benefits of using M-commerce services for all stakeholders. The main challenge faced by the customers in the M-commerce adoption is the convenience of purchase through retail shops as the selection of desired products and immediate purchase becomes easy over there. This study will provide valuable help to the customers to explore the factors that affect their decisions and to help M-commerce service providers to understand critical factors affecting customers’ decisions in the adoption and use of M-commerce services.

### 1.1 Rationale of the study

Most researchers have studied M-commerce by taking the factors such as ease of doing, perceived usefulness, perceived ease of use, satisfaction, technological adoption, security, and trust issues [20, 21]. Researchers also studied the factors such as ease of use and personal and economic factors separately. The present study fills the gap in the available literature by referring to the previous study. No significant study was found related to safety factors during the COVID-19 outbreak. There is a significant gap found by disclosing the ease of doing, economic, and safety factors. Numerous studies have been conducted during the COVID-19 outbreak related to digital payment [17], digital marketing strategy, trust [22] and mobile payment systems [23]. However, no significant study was found on adopting and using M-commerce services during the COVID-19 pandemic. This has motivated us to conceptualize and conduct a study on the adoption and use of M-commerce during the COVID-19 pandemic.

## 2 Literature review

### 2.1 Personal factors

In today’s hectic and highly competitive world, customers are looking for customized services that would help them do their work at their convenient time and place. Considering these in mind, online retailers have been making many changes in their existing interface and services through which personalized features could be available to customers. It would promote ease of using mobile applications, and customers will be satisfied after completing any commercial transactions without fearing insecurity in their minds [24]. Online retailers have been using innovative technology to use mobile commerce efficiently by developing new applications. Various features have been added while designing mobile applications, like personalization, privacy, ubiquity, and many more [25]. Consumers were unwilling to conduct online transactions due to many considerations such as privacy and security, more preference for physical stores in place of online stores for purchase, fear of fraud, and financial losses, which led to a lack of trust among consumers [26]. The most significant concern was to keep consumers’ information highly confidential so that there may be no misuse of their personal information and privacy can be maintained as promised to the consumers by the companies.

The privacy of consumers’ information was considered the top-most priority to be trust between consumers and the companies [27]. In his study, the author identified that consumers had no problem sharing their personal information with third-party companies while using information available at third-party websites during their visit upon searching for information related to their current needs. They were neutral in terms of information privacy. While installing mobile applications, the company’s apps now ask for access to consumers’ contacts, locations, galleries, and other personal details. Once consumers agree to share the company, allow him/her to go further. In this approach, a business can keep track of its customers’ actions and utilize that knowledge to plan its next steps. Even they used to sell the data to third-party service providers [28]. The serious issues of recording consumers’ real-time movement in sharing locations with companies were discovered. However, many companies, such as telecom operators, used to record and analyze their customers’ overall movements as they have complete control over all the information and action of their customers. Hence, privacy has become a significant concern [29]. Consumers needed to adopt M-commerce more quickly due to the nonexistence of trust in the promise made by the companies, as consumers considered it too risky to use M-commerce. There were many issues like consumer information privacy, online mobile transaction safety concerns, reliability of the vendors, and mobile services. All these kept most consumers in a dilemma about whether to use mobile applications or not, as they feared financial loss. Hence, they preferred physical purchases over M-commerce [30, 31].

Apparent utility, trust, and satisfaction observed ease of use of m-commerce prompted the M-commerce adoption (M-commerce services). Consumers had become smarter in shifting from traditional to mobile commerce; the first concern was their safety [32]. Mobile users have significantly increased, consuming the vast bandwidth of mobile internet providers. Users hesitate to use M-commerce because of payment problems, trust, security issues, and complications of the mobile application. M-commerce demands more security than traditional E-commerce [33]. Users were considering E-Commerce safer than M-commerce based on privacy and trust while doing transactions over the internet. Suppose customers get reliable service from online retailers. In that case, there will be trust in the relationship between the company and its customers, which would benefit both of them in the long run [34]. In research conducted in the Malaysian context, perceived ease of use perceived cost, and trust was identified among the factors that affect mobile commerce adoption by Malaysian customers [35]. Trust in technology adoption among customers positively impacted mobile commerce adoption [36, 37]. In a similar study conducted in Ecuador to examine customer intentions toward mobile commerce adoption, it was observed that perceived trust was among the key factors considerably forecasting the consumers’ behavioural intention to adopt and use mobile commerce [38]. However, it was found that consumers’ behavioural intention toward mobile commerce adoption was significantly influenced by the trust as a critical factor [39]. Trust was identified as a key deterring factor for mobile commerce adoption [40]. In light of the COVID-19 pandemic, consumers’ shopping patterns and living standards had a direct impact on consumers’ behavioural intention toward mobile commerce adoption [22].

H1 Personal factors affect the customers’ adoption and use of M-commerce services.

### 2.2 Economic factor

The fear of losing money has made it difficult for consumers to adopt M-commerce services. Yet, numerous M-commerce service providers have attempted to encourage consumers to adopt and use M-commerce services. This would further boost M-commerce transactions; however, this endeavour could have been more successful in positively impacting consumers due to their economic considerations while deciding to use it [41]. Subsequently, M-commerce became a personalized action of consumers that comprised economic transactions, and they were more vigilant with their acceptance of M-commerce services due to economic risk. Hence, they did not blindly embrace collective social practices [42]. However, M-commerce, which has become the contemporary approach to conducting business electronically, might distress the acceptance and use of M-commerce services due to economic factors [43]. In research conducted in the Malaysian context, the perceived financial cost was recognized among the factors positively associated with mobile commerce adoption by Malaysian customers [35]. In addition, customers had expressed their fears about economic issues related to mobile commerce transactions, associated security risks, and transactions’ privacy [40]. However, customers who fit into the low and medium-low socio-economic segments feared losing their money and financial data while using mobile commerce because, due to their poor economic condition, they were not ready to adopt mobile commerce [44].

H2 Awareness of economic factors affects the customers’ adoption and use of M-commerce services.

### 2.3 Ease of doing factors

The author observed a strong relationship between ease of use and trust while using mobile commerce. These apps were too easy to use due to their user-friendly innovative features and reliability of information security, which led to the privacy of consumers’ information. All these developed a strong trust among the consumers towards the company and its products. Mobile apps have made consumers work too quickly based on reviews, ratings, likes, and comments; consumers quickly decide to select and purchase the products they are looking for [45].

In the MENA region, KPMG surveyed to know the usage of wireless networks for mobile banking and found that twenty-seven per cent of users perform transactions through mobile banking. In contrast, fifty per cent were facing problems using smartphones. Hence, there was still a need to create more awareness about mobile devices’ usage in online banking and shopping [46]. Consumers were looking for convenient and reliable tools for online transactions, and if there were safety, trust would be developed among them [47]. One of the elements of social psychology, i.e. ease of use for mobile commerce transactions, was observed as a critical factor positively influencing customers’ intention toward mobile commerce adoption in Ecuador [48]. In another similar study, ease of use was identified as a critical factor influencing customers’ intention toward mobile commerce adoption [49].

Moreover, mobile commerce, an emerging occurrence globally, succeeded in attracting youth worldwide, encouraging them to use and adopt the mobile commerce platform. However, the ease of use and convenience of online shopping through mobile commerce applications were among the key concerns in consumers’ minds [50]. The fear of losing money has made it difficult for consumers to adopt M-commerce services. Yet, numerous M-commerce service providers have attempted to encourage consumers to adopt and use M-commerce services. This would further boost M-commerce transactions; however, this endeavour could have been more successful in positively impacting consumers due to their economic considerations while deciding to use it [41]. Subsequently, M-commerce became a personalized action of consumers that comprised economic transactions, and they were more vigilant with their acceptance of M-commerce services due to economic risk. Hence, they did not blindly embrace collective social practices [42]. However, M-commerce, which has become the contemporary approach to conducting business electronically, might distress the acceptance and use of M-commerce services due to economic factors [43]. In research conducted in the Malaysian context, the perceived financial cost was recognized among the factors positively associated with mobile commerce adoption by Malaysian customers [35]. In addition, customers had expressed their fears about economic issues related to mobile commerce transactions, associated security risks, and transactions’ privacy [40]. However, customers who fit into the low and medium-low socio-economic segments feared losing their money and financial data while using mobile commerce because, due to their poor economic condition, they were not ready to adopt mobile commerce [44].

H3 Ease of doing factors affects the customers’ adoption and use of M-commerce services.

### 2.4 Safety factor

Various elements have been indicated in the previous studies that influence M-commerce adoption among customers worldwide, with perceived security being the essential aspect to consider when determining whether or not to use M-commerce. Safety was the foremost concern among consumers using M-commerce services to complete diverse commercial transactions [51]. The safety of consumers’ personal information from unauthorized access was considered at risk by M-commerce users as they thought that any third-party organizations could use their information for personal gain [41]. Another study also found perceived security among the critical aspects that affect M-commerce services’ acceptance [52]. Additionally, another research study supported safety as the crucial factor impacting M-commerce adoption among consumers who preferred their safety first while purchasing over the internet [53].

Furthermore, M-commerce has transformed the global marketplace. The current study revealed perceived security as the leading significant positive direct association with trust development, trailed by social media influencers. Additionally, the safety factor was observed as the vital forecaster of M-commerce services’ adoption among the Gulf Cooperation Council (GCC) countries’ consumers [54, 55]. Mobile commerce applications have witnessed remarkable growth in their acceptance and adoption during and after COVID-19. However, to withstand customers’ loyalty and trust, the security of mobile commerce applications had a positive perception among consumers’ intention towards mobile commerce adoption [56].

H4 Safety factors during the Covid-19 pandemic increased the adoption and use of M-commerce services among the customers.

Table 1 presents the sources of constructs used in the study and their sources. These constructs were used in the design of the questionnaire.

**Table 1:** Constructs of the study and its sources.

S. No.	Constructs	Sources
1	Personal factor	Vinerean et al., 2022; Tarhini et al., 2019; Pipitwanichakarn and Wongtada, 2018; Verkijika, 2018; Naqvi and Al-Shihi, 2014; Lin et al, 2014; Wei et al, 2009.
2	Economic factor	Dakduk et al., 2020; Naqvi and Al-Shihi, 2014.
3	Ease of doing	Salamah et al, 2022; Petcharat and Leelasantitham, 2021; Peña-García et al, 2019.
4	Safety factors during the COVID-19 pandemic	Gull et al., 2022.

During the Covid-19 pandemic, this study aims to address a substantial knowledge vacuum by shedding light on the influence of individual characteristics, simplicity of usage, understanding of economic factors, and safety factors on the adoption and use of M-commerce services.

## 3 Objectives of the study

The following are the main objectives of this study:

1. To examine the effect of personal factors on the adoption and use of M-commerce services.
2. To study the impact of economic factors on the adoption and use of M-commerce services.
3. To analyze the impact of ease of doing factors on the adoption and use of M-commerce services.
4. To examine the effect of safety factors during the Covid-19 pandemic on the adoption and use of M-commerce services.

## 4 The research model

**Figure 1:**
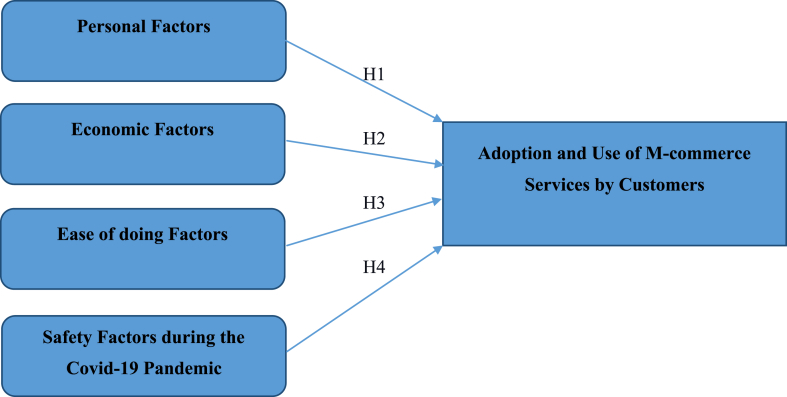
Research model.

Figure 1 depicts the research model developed in accordance with the study’s objectives.

## 5 Research method

This research aims to analyze the impact of personal, economic, and ease of doing factors and safety factors in customer adoption and use of M-commerce services. A mixed-method approach was used in this research to collect the data from primary and secondary sources. 340 customers were surveyed through a questionnaire to obtain their responses to these factors. Cronbach’s Alpha was used for reliability analysis. The data were analyzed using descriptive statistics and correlation analysis. Hypotheses were tested by applying ANOVA and bivariate regression analysis.

### 5.1 Data collection and sampling

Both primary and secondary sources were used to collect the data for the present study. The research instrument used in this study was a structured questionnaire. The questionnaire was designed with the help of the available literature review. The questionnaire consists of 26 statement-based questions focusing on five factors used in the study. For measuring the responses, a five-point Likert scale was used where (1) Strongly disagree; (2) Disagree; (3) Neither agree nor disagree; (4) Agree; (5) Strongly agree. The secondary sources used in the study were research papers, journals, business magazines, and published literature. Data was collected using the convenience sampling approach, one of the non-probability sampling techniques. Before distributing the questionnaire, a pilot test was conducted with 40 respondents in order to collect their feedback, confirm the questionnaire’s simplicity, readability, and understanding and develop the questionnaire more effectively. The 40 respondents provided positive feedback, and the pilot test results confirmed that the questionnaire was clear to the selected respondents. The final questionnaire was then distributed among the respondents. A sample of 340 respondents was selected who were either familiar with the M-commerce concept or were using the M-commerce services. Informed consent was obtained from all participants for the research.

### 5.2 Data analysis

The SPSS was used to analyze the data gathered for this research. Numerous statistical tools were used to analyze the data. Descriptive analysis was used to present the percentages and frequencies of the respondent’s demographic profiles. The check the relationship among the variables, the correlation was determined. For hypothesis testing, regression and ANOVA were used.

#### 5.2.1 Demographic profile

Table 2 provides the demographic information of the respondent. The sample survey represents that most respondents have aged less than 26–30 years. 77.35% of respondents are male, and 22.65% are female. Most respondents either have a bachelor’s degree or master’s degree or are PhD holders. The majority of respondents are private employees. All respondents use smartphones and use M-commerce websites to purchase goods and services. Most respondents have been using M-commerce services for less than one year to 5 years. 87% of customers prefer M-commerce and retail shops to purchase goods and services.

**Table 2:** Demographic profile of the respondents.

Demographic Variable	Frequency (n)	Percentage (%)
**Age**		
Less than 20	16	4.71
21–25	75	22.06
26–30	116	34.12
31–35	75	22.06
36–40	50	14.71
Above 40	8	2.35
**Gender**		
Male	263	77.35
Female	77	22.65
**Educational Level**		
Diploma	25	7.35
Bachelor’s degree	160	47.06
Master’s degree	120	35.29
Ph.D	35	10.29
**Profession**		
Government Employee	90	26.47
Private Employee	190	55.88
Business	15	4.41
Others	45	13.24
**Do you use Smart Phone?**		
Yes	340	100
No	0	0
**Do you use any M-commerce website for online purchase of goods and services?**		
Yes	340	100
No	0	0
**If yes, how long have you been using M-commerce for your online purchasing?**		
Less than 1 year	141	41.47
1–5 years	183	53.82
More than 5 years	16	4.71
**What do you prefer for your purchasing?**		
Traditional purchase (from retail shops or Malls)	28	8.24
M-commerce	17	5
Both	295	86.76

The sample survey also revealed that electronic items are the first choice of customers in an online purchases, followed by apparel and groceries. Kitchen appliances and surgical products are in fourth and fifth place, respectively, in order of preference.

#### 5.2.2 Descriptive statistics

According to the values of mean, minimum, maximum values and standard deviation in Table 3, it is revealed that all four variables have a significant impact on the adoption and use of M-commerce by customers. However, compared to the other variables, the Covid-19 pandemic factors significantly impact the adoption and use of M-commerce services.

**Table 3:** Descriptive statistics of the variables.

	N	Minimum	Maximum	Mean	Std. Deviation
Personal Factors	340	2.66	4.58	3.80	.47
Economic Factors	340	2.40	5.00	4.04	.51
Ease of doing Factors	340	2.50	5.00	3.83	.60
Covid-19 Pandemic Factors	340	3.00	5.00	4.47	.52

#### 5.2.3 Reliability test

The reliability analysis (Cronbach’s alpha) was used to examine the internal consistency of the constructs used in the study. If Likert-type scales were employed in the research, it is crucial to report Cronbach’s alpha as a measure of the scales’ internal consistency reliability [57]. The reliability measure was calculated with the help of SPSS. Table 4 outlines the dependability of each construct and its interpretations. According to Cronbach’s alpha, the data obtained had an internal consistency and reliability that ranged from .835 to .919. This indicates that the data had high internal consistency and reliability.

**Table 4:** Reliability analysis of the variables.

Constructs	N	Number Of Items	Cronbach’s Alpha	Internal Consistency
Personal Factors	340	7	0.919	Excellent
Economic Factors	340	6	0.835	Excellent
Ease of doing Factors	340	7	0.895	Excellent
Covid-19 Pandemic Factors	340	6	0.845	Excellent

#### 5.2.4 Correlation analysis

The correlation analysis data on the relationship between the variables found in the adoption and use of the M-commerce services scale are shown in Table 5. Pearson’s correlation coefficient is commonly used to compare the outcomes of tests [58]. The value of the correlation coefficient should be less than 0.7 to be deemed suitable [59].

**Table 5:** Correlation analysis of the variables.

	PF	EF	EODF	C19F	A&U	p value
Personal Factors	1					.000
Economic Factors	0.425645946	1				.000
Ease of doing Factors	0.506634622	0.516502403	1			.000
Covid-19 Pandemic Factors	0.604706399	0.378735752	0.497364137	1		.000
Adoption and use of M-commerce by customers	0.286493776	0.50050458	0.570525958	0.329742154	1	.000

A strong correlation was found between personal factors r (340) = .29, p < 0.05, economic factors r (340) = .50, p < 0.05, ease of doing factors r (340) = .57, p < 0.05, Covid-19 pandemic factors r (340) = .32, p < 0.05 and adoption and use of M-commerce services by the customers.

#### 5.2.5 Bivariate regression analysis

This study consists of a dependent variable (Adoption and use of M-commerce by customers) and four independent variables (Personal Factors, Economic Factors, Ease of doing Factors, and Covid-19 Pandemic Factors). Bivariate regression analysis was used because of the possibility of multicollinearity and the need for individual analysis of regression coefficients.

Table 6 shows the standard deviation-model summary estimation, which reveals that the first independent variable, personal factors, is a significant predictor in customer adoption and use of M-commerce. It has a significant variance in the value of the adoption of M-commerce by customers.

**Table 6:** Estimation of the standard deviation- Model Summary (Independent Variable- Personal Factors).

Theoretical Form of Model						
Customer’s Satisfaction = a + b Personal Factors						
Regression Model Summary^b^ 1						
Model	R	R Square	Adjusted R Square	Std. Error of the Estimate	F Change	Significance F
1	0.495	0.245	0.243	0.896	109.820	.000

^a^Predictors: (Constant), Personal Factors.

b Dependent Variable: Adoption and use of M-commerce by customers.

The model is also significant based on the values of F, p, and R^2^.

According to Table 7, the estimation of the standard deviation-model summary-the second independent variable, awareness of economic factors, is a significant predictor of customer adoption and M-commerce use. It has a significant variance in the value of the adoption of M-commerce by customers. The model is also significant based on the values of F, p, and R^2^.

**Table 7:** Estimation of the standard deviation- Model Summary (Independent Variable- Economic Factors).

Theoretical Form of Model						
Customer’s Satisfaction = a + b Economic Factors						
Regression Model Summary^b^ 2						
Model	R	R Square	Adjusted R Square	Std. Error of the Estimate	F Change	Significance F
2	0.560	0.313	0.311	0.854	154.594	.000

^a^Predictors: (Constant), Economic Factors.

b Dependent Variable: Adoption and use of M-commerce by customers.

Table 8 indicates the estimation of the standard deviation-model summary, which shows that the third independent variable, the ease of doing factor, is a significant predictor in customer adoption and use of M-commerce. It has a significant variance in the value of the adoption of M-commerce by customers. The model is also significant based on the values of F, p, and R^2^.

**Table 8:** Estimation of the standard deviation- Model Summary (Independent Variable- Ease of doing Factors).

Theoretical Form of Model						
Customer’s Satisfaction = a + b Ease of doing Factors						
Regression Model Summary^b^ 3						
Model	R	R Square	Adjusted R Square	Std. Error of the Estimate	F Change	Significance F
3	0.572	0.327	0.325	0.846	164.651	.000

^a^Predictors: (Constant), Ease of doing Factors.

b Dependent Variable: Adoption and use of M-commerce by customers.

Table 9 represents the estimation of the standard deviation-model summary, which reveals that the fourth independent variable, personal factors, is a significant predictor in customer adoption and use of M-commerce. It has a significant variance in the value of the adoption of M-commerce by customers. The model is also significant based on the values of F, p, and R^2^.

**Table 9:** Estimation of the standard deviation- Model Summary (Independent Variable- Safety factors during Covid-19 pandemic).

Theoretical Form of Model						
Customer’s Satisfaction = a + b Safety factors during Covid-19 Pandemic						
Regression Model Summary^b^ 4						
Model	R	R Square	Adjusted R Square	Std. Error of the Estimate	F Change	Significance F
4	0.636	0.405	0.403	0.795	230.645	.000

^a^Predictors: (Constant), Safety factors during Covid-19 Pandemic.

b Dependent Variable: Adoption and use of M-commerce by customers.

#### 5.2.6 Testing of hypotheses

The ANOVA of each of the four regression predictor models is summarized in Table 10. ANOVA model was used to evaluate the association between the models across all variables. The ANOVA can assist in the determination of whether or not the means of independent variables differ significantly. Because once we understand that each independent variable’s mean differs from the other, we can determine which relates to our dependent variable.

**Table 10:** Variation analysis of the Variables - ANOVA.

ANOVA^a^						
Model		Sum of Squares	Df	Mean Square	F	Sig.
1	Regression	88.286	1	88.286	109.820	.000^b^
	Residual	271.723	338	0.803		
	Total	360.009	339			
2	Regression	112.984	1	112.984	154.594	.000^c^
	Residual	247.025	338	0.730		
	Total	360.009	339			
3	Regression	117.926	1	117.926	164.651	.000^d^
	Residual	242.082	338	0.716		
	Total	360.009	339			
4	Regression	146.021	1	146.021	230.645	.000^e^
	Residual	213.988	338	0.633		
	Total	360.009	339			

a Dependent Variable: Adoption and use of M-commerce by customers.

b Predictors: (Constant), Personal Factors.

c Predictors: (Constant), Economic Factors.

d Predictors: (Constant), Ease of doing Factors.

e Safety factors during Covid-19 Pandemic.

The values of coefficients regression of the four variables are presented in Table 11. It is evident from the values of Beta, t, and p that all four independent variables significantly predicted the adoption and use of M-commerce by customers. This signifies that when customers plan to purchase their goods and services using M-commerce services, their decisions are affected by various personal factors, ease of doing factors, economic factors, and safety-related factors concerning Covid-19 safety measurements. The result significantly supports the association and impact of these factors in customers’ adoption and use of M-commerce.

**Table 11:** Coefficients regression of the variables.

Model	Unstandardized Coefficients		Standardized Coefficients	t	Sig.
	B	Std. Error	Beta		
(Constant)	0.600	0.177		3.385	.000
Personal Factors	0.275	0.048	0.536	10.479	.000
Economic Factors	0.203	0.054	0.601	12.433	.000
Ease of doing Factors	0.072	0.056	0.596	12.831	.000
Safety factors during Covid-19 Pandemic	0.475	0.050	0.660	15.187	.000

^a^Dependent Variable: Adoption and use of M-commerce by customers.

Following results have been drawn with the help of p values, indicated in Table 8:

H1 Personal factors affect the customers’ adoption and use of M-commerce services.

Hypothesis 1 is accepted. This signifies that personal factors affect M-commerce use and adoption among customers.

H2 Awareness of economic factors affects the customers’ adoption and use of M-commerce services.

Hypothesis 2 is accepted. This signifies that economic factors affect M-commerce use and adoption among customers.

H3 Ease of doing factors affects the customers’ adoption and use of M-commerce services.

Hypothesis 3 is accepted. This signifies that the ease of doing factors affect M-commerce use and adoption among customers.

H4 Safety factors during the Covid-19 pandemic increased the adoption and use of M-commerce services among the customers.

Hypothesis 4 is accepted. This signifies that the safety factors during the Covid 19 pandemic affect M-commerce use and customer adoption. The customers preferred adopting and using M-commerce services during this period to purchase their goods and services because of safety issues.

## 6 Results and discussion

This study suggests that the use of M-commerce services and their adoption among the customer is primarily affected by personal factors, economic factors, ease of doing factors, and safety factors during the Covid-19 pandemic. Based on the study results, it has been observed that the adoption and use of M-commerce are familiar among customers, but some factors still affect this decision. Description statistics show that decision regarding M-commerce use and adoption among customers is significantly affected by all four independent variables.

Hypothesis 1 was accepted in the study and signifies that personal factors affect M-commerce use and adoption among customers. Consumers’ buying behaviour patterns and living standards had a direct impact on consumers’ behavioural intention toward mobile commerce adoption [22]. Lack of trust and security risk are essential factors influencing M-commerce use and adoption [60].

Hypothesis 2 was accepted in the study and signifies that economic factors affect M-commerce use and adoption among customers. According to this survey, people are also of the view that m-commerce makes a substantial contribution to the growth of the economy. M-commerce can benefit enterprises in various forms, such as increased revenue, market reach, two-way communication between customers and suppliers, enhanced employee productivity, quick transaction process and increased company brand image [61]. It is also identified in an imperial study that greater E-commerce adoption leads to more significant profit [62]. It contributes significantly to the growth of numerous other related industries, such as packing services, courier services, and banking. M-commerce benefits merchandisers since it boosts sales prospects.

Hypothesis 3 was accepted in the study and signifies that the ease of doing factors affect M-commerce use and adoption among customers. The study’s findings suggested that M-commerce offers a flexible approach to buying goods and services compared to traditional purchases. Compared to traditional purchases, M-commerce purchases are more flexible and easy because they can be done anytime. Smartphones or wireless devices have enhanced the mobility and accessibility of information, contributing various advantages such as ease of use, swiftness and convenience [63, 64]. Ease of doing is critical in attracting consumer intention towards M-commerce adoption [48]. M-commerce makes it simple to buy things online since it makes them more readily available than in physical stores. Customers often prefer shopping online since the deals and discounts they find on the M-commerce website entice them. They prefer to buy products and services online since they are delivered swiftly. Customers also prefer to buy goods and services online since they can get all the stuff they need in the amount and quantity they want.

Hypothesis 4 was accepted in the study and signified that the safety factors during Covid 19 pandemic affected M-commerce use and adoption among customers. A significant outcome of the present study is that the customers opted to buy the products through M-commerce during the COVID-19 pandemic because of social isolation and health measurement standards. Due to the possibility of contracting the virus, they decided that shopping online was safer than going to a mall or neighbouring store. An empirical study found that safety considers a key factor while purchasing goods and services through the internet [53]. The COVID-19 pandemic significantly modified consumer buying behaviour. Consumer buying behaviour routines shifted from traditional to online purchases, such as the safety of home deliveries or in-store pickup and digital payment [65]. The study’s findings also revealed that people adopt mobile commerce more frequently due to the danger of contracting an infection when visiting a mall or retail establishment.

Based on the results, the M-commerce companies are advised to increase the awareness of the customers towards the economic benefits of using M-commerce services and should consider the security factors, delivery factors, availability of products and services, and customer preferences that affect the customer’s decision. Companies should also increase their prompt delivery services as customers prefer to purchase online when goods are delivered quickly. Companies should also strengthen the services of easy and prompt returns and find out how the customer’s time could be saved in search of required goods and services per their demand.

## 7 Implications of the study

The present study will significantly help M-commerce customers understand the importance of using M-commerce services during pandemic times. The study outcomes will also benefit the companies and industries involved in such services in understanding the factors that prevent customers from using M-commerce services frequently. As per the results, if companies work on these factors, there will be a massive increase in the customer base of all such companies. This will directly contribute to the economic development of a country.

## 8 Conclusions

Some key conclusions might be derived from the study’s findings and analysis. The results showed that all four factors have a significant role in customer adoption and use of M-commerce services. Customers’ decisions are affected mainly by economic factors and the Covid-19 pandemic factors compared to personal and ease of doing factors. According to ANOVA, these factors’ influence on customers’ decision-making process differed, but all factors positively and negatively impacted their purchasing decisions. Due to various health and safety measurements, M-commerce services have significantly increased during this pandemic. Customers preferred to purchase goods and services during this period to safeguard them from getting infected by visiting retail shops and malls. A positive correlation among all the variables revealed a significant association. The significance level is high in two variables, economic factors, and ease of doing factors. It can be concluded that customers are aware of M-commerce services and frequently use them to purchase their goods and services, but it is less frequent than in the case of purchases through nearby retail shops or malls. Customers feel that it is easy to access the purchase required goods and services through M-commerce. However, their decisions, use, and adoption are greatly affected due to security and risk factors of stolen data involved in the M-commerce purchase. On the other hand, the customer also believes that M-commerce services are slightly time-consuming.

This study helps to clarify four crucial factors that influence the adoption and use of M-commerce services among customers. However, one of the main limitations of the study is that it is based on some selective variables in the adoption and use of M-commerce. Future studies could examine how different cultural variables affect M-commerce adoption and use while considering present and other relevant factors.

## Declarations

### Author contribution statement

Mohammad Wasiq; Amar Johri: Conceived and designed the experiments; Performed the experiments; Analyzed and interpreted the data; Contributed reagents, materials, analysis tools or data; Wrote the paper.

Prakash Singh: Conceived and designed the experiments; Wrote the paper.

### Funding statement

This work was supported by Deputyship for Research & Innovation, Ministry of Education in Saudi Arabia through the project number 8032.

### Data availability statement

Data included in article/supp. material/referenced in article.

### Declaration of interest’s statement

The authors declare no competing interests.

### Additional information

Supplementary content related to this article has been published online at https://doi.org/10.1016/j.heliyon.2022.e12532.

## Appendix A Supplementary data

QUESTIONNAIRE.pdf QUESTIONNAIRE.pdf
