# Supplementary material for: Factors affecting adoption and use of M-commerce services among the customers in Saudi Arabia
Source: Heliyon. 2022 Dec 22;8(12):e12532. doi: 10.1016/j.heliyon.2022.e12532 (PMC9834744; doi:10.1016/j.heliyon.2022.e12532)
Supplement: QUESTIONNAIRE.pdf [file mmc1.pdf]

Questionnaire on M-Commerce

Section 1:

Age

- Less than 20
- 21-25
- 26-30
- 31-40
- Above 40

Gender:

- Male
- Female
- Prefer not to say

Educational Level:

- High School
- Diploma
- Bachelor’s degree
- Master’s degree
- P.hD
- Other

Profession:

- Government
- Private
- Business

Section 1

Do you use Smart phone

- Yes
- No

Do you use any mobile commerce website for online purchase?

- Yes
- No

If yes, how long have you been using m-commerce for your online purchasing?

- 1-5 years
- 6-10 years
- More than 10 years

What do you prefer for your purchasing?

- Traditional purchase
- E-commerce/ M-commerce
- Both

In case of online purchase, what is your preference?

- Groceries
- Electronic
- Apparels
- Kitchen Appliances
- Surgical products
- Other________________________

Section 2

5 point scale:-

1. **Strongly disagree**
2. **Disagree**
3. **Neutral**
4. **Agree**
5. **Strongly agree**

1. M-commerce reduces job opportunities by reducing labor force.
2. M-commerce provides significant contribution in the economic development of the economy.
3. M-commerce provides significant contribution in the development of various other associated sectors such as packing services, courier services etc .
4. M-commerce increases sales opportunity thus it is beneficial for merchandisers.
5. M-commerce is also beneficial from financial perspectives.

6. M commerce is flexible for purchasing the goods and services rather than traditional purchase.
7. Traditional purchase is more flexible for purchasing the goods and services rather than M-commerce purchase.
8. Purchasing through M-commerce is convenient as compare to traditional purchase and could be done anytime.
9. M commerce is an easy way of purchasing the material online.
10. I found all the goods and services easily through the use of M-commerce as compare to retails shops.
11. Do you feel that your online purchase is affected because of speed of internet or connection problem?
12. It is easy to access the purchase of required goods and services through M-commerce as compare to traditional purchasing.
13. You normally hesitate to purchase the goods and services online due to multiple security and risk factors.
14. You prefer to use traditional purchase as compare to M-commerce because you receive prompt delivery of the product in traditional purchase.
15. Selection of goods and services to be bought is easy in traditional purchase as compare to M-commerce.
16. M-commerce purchase is time consuming as compare to traditional purchase.
17. You prefer purchasing online because you are attracted by the offers and discounts that you receive on M-commerce website.
18. You have full trust on purchasing goods and services on your phone.

19. You prefer cash on delivery instead of paying through debit or credit card because of security measures
20. You prefer not to purchase through M-commerce website as your data might be stolen
21. You prefer to purchase something online when on M-commerce vendor promises to provide you easy and prompt returns.
22. You prefer to purchase goods and services online when it is to be delivered quickly
23. You prefer to purchase goods and services online when all you required product available as per your desired quantity and quality.
24. During COVID 19 Pandemic period, you prefer to purchase through M-commerce because of social distancing and health measurement guidelines.
25. You found it safer to do your purchase online rather than visiting a mall or a shop nearby because of the risk of getting infected.
26. Your frequency of using M-commerce has increased because of risk of getting infected by visiting a retail shop or a mall.
